# Quality and Accountability of ChatGPT in Health Care in Low- and Middle-Income Countries: Simulated Patient Study

**DOI:** 10.2196/56121

**Published:** 2024-09-09

**Authors:** Yafei Si, Yuyi Yang, Xi Wang, Jiaqi Zu, Xi Chen, Xiaojing Fan, Ruopeng An, Sen Gong

**Affiliations:** 1 UNSW Business School and CEPAR The University of New South Wales Kensington Australia; 2 Division of Computational and Data Sciences Washington University in St Louis St. Louis, MO United States; 3 Brown School Washington University in St Louis St Louis, MT United States; 4 Global Health Research Center Duke Kunshan University Kunshan China; 5 Department of Health Policy and Management Yale University New Haven, CT United States; 6 Department of Economics Yale University New Haven, CT United States; 7 School of Public Policy and Administration Xi’an Jiaotong University Xi'an China; 8 Silver School of Social Work New York University New York, NY United States; 9 Centre for International Studies on Development and Governance Zhejiang University Hangzhou China

**Keywords:** ChatGPT, generative AI, simulated patient, health care, quality and safety, low- and middle-income countries, quality, LMIC, patient study, effectiveness, reliability, medication prescription, prescription, noncommunicable diseases, AI integration, AI, artificial intelligence

## Abstract

Using simulated patients to mimic 9 established noncommunicable and infectious diseases, we assessed ChatGPT’s performance in treatment recommendations for common diseases in low- and middle-income countries. ChatGPT had a high level of accuracy in both correct diagnoses (20/27, 74%) and medication prescriptions (22/27, 82%) but a concerning level of unnecessary or harmful medications (23/27, 85%) even with correct diagnoses. ChatGPT performed better in managing noncommunicable diseases than infectious ones. These results highlight the need for cautious AI integration in health care systems to ensure quality and safety.

## Introduction

The rise of generative artificial intelligence (AI) models like ChatGPT is transforming the health care landscape, especially in low- and middle-income countries (LMICs). These regions, often facing shortages of health care professionals, are increasingly turning to AI tools for medical consultation, aided by growing internet and smartphone access [1]. Research has highlighted generative AI use in the fields of cardiology [2] and orthopedic diseases [3]. However, there are concerns about the accuracy and safety of AI models like ChatGPT [4] given their lack of legal or professional accountability. This is crucial in medical settings, where precise and reliable decision-making is vital. Our study focuses on assessing ChatGPT’s performance in treatment recommendations for common diseases in LMICs, addressing a critical need for the responsible application of AI in health care.

## Methods

### Overview

We used the simulated patient (SP) method to create a realistic testing environment for ChatGPT with GPT-3.5 from August 8 to 19, 2023. SPs are healthy individuals trained to consistently mimic real patients and their symptoms [5]. We trained the SPs to present 9 common, previously validated diseases [5-8]. We asked ChatGPT to act as a doctor in an LMIC and offer consultations. The SPs detailed their primary concerns, gave standardized responses to every question, and recorded all diagnoses and medication recommendations, which were cross-referenced with clinical guidelines to assess their accuracy and appropriateness. For a robust analysis, we presented each disease to ChatGPT 3 times. We conducted descriptive analyses with the final sample of 27 independent trials.

### Ethical Considerations

The Ethics Committee of the First Affiliated Hospital of Xi’an Jiaotong University approved the study (LLSBPJ-2024-WT-019).

## Results

**Figure 1:**
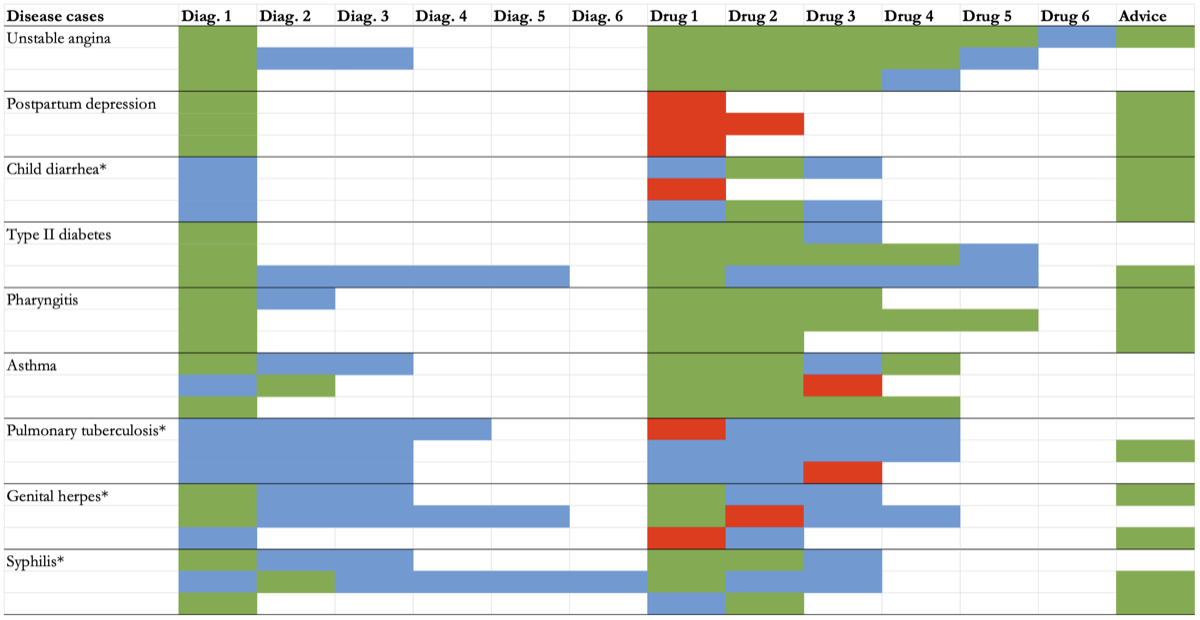
Heatmap comparing ChatGPT’s responses with clinical guidelines. The asterisks (*) indicate infectious diseases; green cells denote correct or appropriate diagnoses or drug prescriptions; blue cells denote incorrect or unnecessary diagnoses or drug prescriptions; and red cells denote harmful drug prescriptions. Each row represents an independent trial.

**Figure 2:**
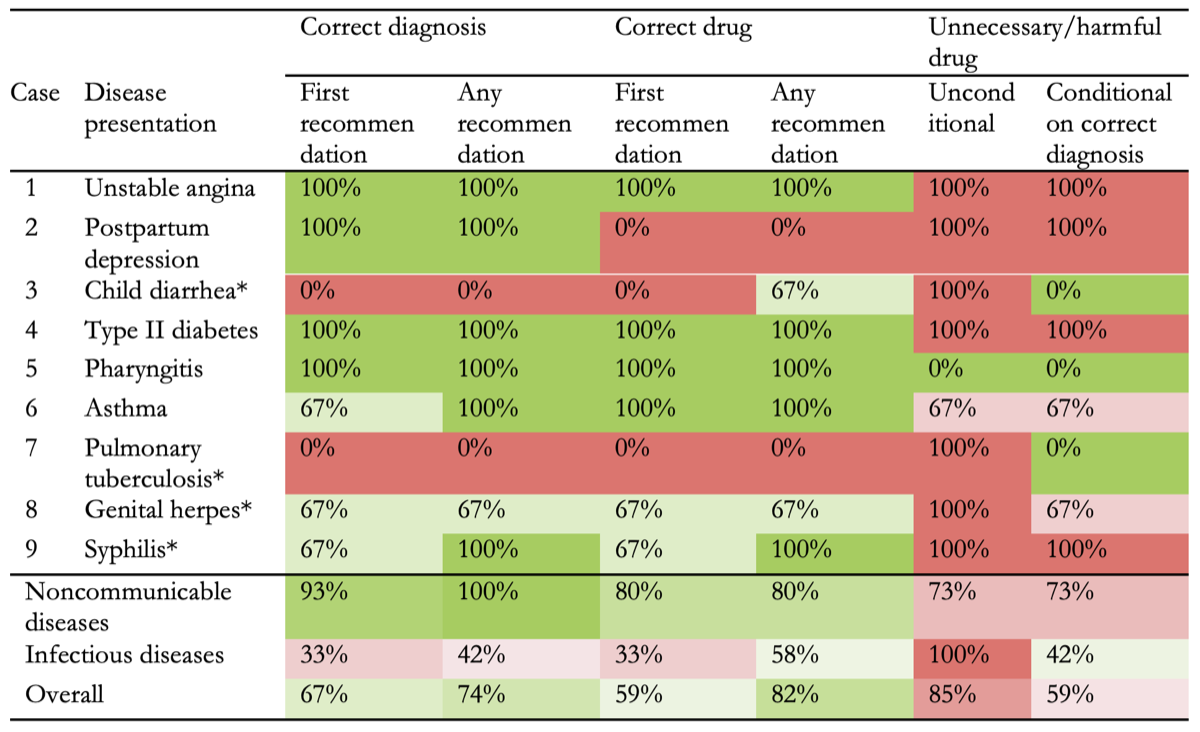
ChatGPT’s capability in diagnosing and treating 9 common diseases. The asterisks (*) indicate infectious diseases; green denotes socially desired outcomes; red denotes undesired outcomes; darker colors denote higher probabilities.

Surprisingly, ChatGPT’s performance varied across trials for each disease (Figure 1). When aggregating the results (Figure 2), ChatGPT had a 67% (18/27) success rate in initial diagnoses and a 59% (16/27) success rate in medication recommendations. When considering all recommendations, these rates increased to 74% (20/27) for any correct diagnosis and 82% (22/27) for any appropriate medication recommendation. However, there was a high rate of unnecessary or harmful medication suggestions, occurring in 85% (23/27) of trials overall and in 59% (16/27) of trials after a correct diagnosis. Our study also highlighted ChatGPT’s varying performance across different types of diseases. Specifically, the AI demonstrated a superior ability in handling noncommunicable diseases compared to infectious diseases, both in terms of diagnosis and medication recommendations.

## Discussion

Our findings reveal a high level of accuracy in both correct diagnoses (74%) and medication recommendations (82%) by ChatGPT. Previous studies using the SP method found that primary care providers in LMICs like China, India, and Kenya could only reach correct diagnoses in 12%-52% of SP visits [5,6]. Therefore, ChatGPT can potentially outperform traditional primary care providers in LMICs in diagnostic accuracy. Since ChatGPT with GPT-3.5 is free, the AI tool has the potential to offer affordable and far-reaching solutions in LMICs, particularly in rural and underserved areas.

However, ChatGPT tended to suggest more unnecessary or even harmful medications (in 85% of trials) than primary care providers (28%-64%) [5,6]. AI models work by analyzing available data using machine learning and deep learning techniques [9]. Their approach to drug prescription can be aggressive due to a lack of professional accountability or a motive to reduce medical expenses. ChatGPT also performed better in managing noncommunicable diseases than infectious diseases. This could be because more information on the former is available for AI training during development [10]. ChatGPT’s performance also varied within each disease case, contrary to our expectation that this would be more standardized.

We acknowledge several limitations. First, a broader array of diseases, especially those specific to different regions, should be used in future studies. Second, we did not introduce more details (ie, location) to avoid the prompts becoming overcomplicated, and by default, ChatGPT’s responses reflect the average population to increase its generalizability. Third, we did not account for the relative importance of the AI’s questions and emotional communications. Fourth, a larger sample size may have enabled us to perform head-to-head comparisons between AI care and traditional care.

Despite the limitations, we present the first audit-study evidence to evaluate ChatGPT’s performance in diagnosing and treating common diseases in LMICs. A rich set of 9 established diseases makes our findings highly relevant to and widely applicable in LMICs. ChatGPT reaches high levels of accuracy in diagnosis and medication recommendations, but also recommends a concerning level of unnecessary or harmful medications. Integrating AI tools like ChatGPT into health care systems in LMICs may potentially improve diagnostic accuracy but also raises concerns about care safety.

## Abbreviations

AI: artificial intelligence
LMIC: low- and middle-income country
SP: simulated patient

## Data Availability

All data generated or analyzed during this study are included in this published article.

## References

[1] Howarth J How many people own smartphones? (2023-2028). Exploding Topics.

[2] Sarraju A, Bruemmer D, Van Iterson E, Cho L, Rodriguez F, Laffin L (2023). Appropriateness of cardiovascular disease prevention recommendations obtained from a popular online chat-based artificial intelligence model. JAMA.

[3] Kuroiwa T, Sarcon A, Ibara T, Yamada E, Yamamoto A, Tsukamoto K, Fujita K (2023). The potential of ChatGPT as a self-diagnostic tool in common orthopedic diseases: exploratory study. J Med Internet Res.

[4] Wang C, Liu S, Yang H, Guo J, Wu Y, Liu J (2023). Ethical considerations of Using ChatGPT in health care. J Med Internet Res.

[5] Kwan A, Daniels B, Bergkvist S, Das V, Pai M, Das J (2019). Use of standardised patients for healthcare quality research in low- and middle-income countries. BMJ Glob Health.

[6] Si Y, Bateman H, Chen S, Hanewald K, Li B, Su M, Zhou Z (2023). Quantifying the financial impact of overuse in primary care in China: a standardised patient study. Soc Sci Med.

[7] Xue H, D’Souza K, Fang Y, Si Y, Liao H, Qin WA, Yip W, Xu D, Gong W, Chen W, Tian J, Tang W, Sylvia S Direct-to-consumer telemedicine platforms in China: a national market survey and quality evaluation. Preprints with The Lancet.

[8] Si Y, Xue H, Liao H, Xie Y, Xu D, Smith M, Yip W, Cheng W, Tian J, Tang W, Sylvia Sean (2024). The quality of telemedicine consultations for sexually transmitted infections in China. Health Policy Plan.

[9] Sellamuthu S, Vaddadi S, Venkata S, Petwal H, Hosur R, Mandala V, Dhanapal R, singh J (2023). AI-based recommendation model for effective decision to maximise ROI. Soft Comput.

[10] Sanders JW, Fuhrer GS, Johnson MD, Riddle MS (2008). The epidemiological transition: the current status of infectious diseases in the developed world versus the developing world. Sci Prog.

